# Lack of Effectiveness of Tramadol for Earlier Limb Weight-Bearing After Femoral Head and Neck Excision in 38 Dogs

**DOI:** 10.3390/ani16071064

**Published:** 2026-03-31

**Authors:** Androniki Krystalli, George M. Kazakos, Ioannis Savvas, Nikitas N. Prassinos

**Affiliations:** 1 Surgery & Obstetrics Unit, Companion Animal Clinic, School of Veterinary Medicine, Faculty of Health Sciences, Aristotle University, 54627 Thessaloniki, Greece; ngreen@vet.auth.gr; 2 Anesthesia and Intensive Care Unit, Companion Animal Clinic, School of Veterinary Medicine, Faculty of Health Sciences, Aristotle University, 54627 Thessaloniki, Greece; gkdvm@vet.auth.gr (G.M.K.); isavas@vet.auth.gr (I.S.)

**Keywords:** canine, coxofemoral joint, opioid analgesics, robenacoxib

## Abstract

The objective of this study was to evaluate, in client-owned dogs, the postoperative analgesic efficacy of the centrally acting analgesic tramadol following femoral head and neck excision (FHNE). Thirty-eight dogs were diagnosed with coxofemoral disease and underwent FHNE. The sample was randomly divided into two groups. Postoperatively, dogs in Group A received robenacoxib (2 mg kg^−1^ bodyweight, sid, per os) until they achieved final weight-bearing of the operated limb, whereas dogs in Group B received the same regimen of robenacoxib in combination with tramadol (5 mg kg^−1^ bodyweight, tid, per os) for fifteen days. All animals were premedicated, sedated, and anesthetized following the same standardized protocol to ensure consistency between groups. Postoperative evaluations were conducted on days 15 and 30, and subsequently at monthly intervals, until full weight-bearing of the affected limb was achieved. Clinical assessments included observation of pain-related behaviors, lameness scoring, and time to initial weight-bearing (TIWB) and time to final weight-bearing (TFWB). Data normality was assessed using the Shapiro–Wilk test, and differences between groups were evaluated using the independent samples *T*-test (*p* = 0.05). Statistical analysis revealed no significant differences between the two groups regarding time to initial (*p* = 0.087) or final weight-bearing. However, a moderate effect size (*d* = 0.570) was observed for TIWB, suggesting a clinically interesting trend toward faster early recovery in the multimodal group. These findings indicate that while adding tramadol did not confer a statistically significant additional advantage in this study, the observed clinical trend warrants further investigation in larger, higher-powered cohorts to determine its definitive clinical impact.

## Simple Summary

This study investigated the potential additive analgesic effect of tramadol in dogs undergoing femoral head and neck excision (FHNE) for coxofemoral joint disease. Thirty-eight client-owned dogs were enrolled and assigned to two postoperative treatment groups. Group A received robenacoxib (2 mg kg^−1^ bodyweight, sid) until full weight-bearing was achieved, while Group B received the same regimen of robenacoxib in combination with tramadol (5 mg kg^−1^ bodyweight, tid) for 15 days. All patients underwent standardized premedication, sedation, and anesthesia protocols. Postoperative evaluations were conducted on days 15 and 30 and, subsequently, at monthly intervals, to assess limb function and weight-bearing progression. The addition of tramadol did not result in a significant difference in either the time to initial or final weight-bearing. These findings suggest that tramadol, at the administered dose and duration, does not confer additional clinical benefit in postoperative pain management following FHNE in dogs.

## 1. Introduction

Pain in veterinary medicine is characterized as a distressing sensory and emotional experience that corresponds to, or mimics the effects of, existing or threatened tissue injury [1]. Severe postoperative pain is a common consequence in dogs undergoing orthopedic surgeries, especially when extensive tissue dissection or invasive fixation is required [2,3,4]. Postoperative orthopedic pain typically has an acute onset and comprises both inflammatory and somatic components, involving soft tissue and bone, in addition to any persistent pain resulting from the underlying orthopedic condition [5].

Acute pain induces significant alterations in metabolic and hemodynamic systems, activates the inflammatory cascade, and elicits a sympathetic stress response [6]. If inadequately managed, these physiological changes can delay healing, increase the risk of complications, and negatively impact the patient’s overall recovery. Accurate pain assessment and treatment using a multimodal protocol are, therefore, considered essential to enhance recovery, improve quality of life, and expedite the return to normal function.

Management of acute pain commonly involves systemic administration of opioids and non-steroidal anti-inflammatory drugs (NSAIDs) [7,8,9]. Among opioids, morphine, methadone, and tramadol are the most frequently used in small-animal practice [10,11]. These agents are effective for mild-to-severe pain, whether acute or chronic, and can reduce perioperative pain while promoting faster postoperative recovery [12].

Tramadol is a centrally acting, atypical, synthetic opioid with two distinct mechanisms of antinociception. Its opioid-mediated effect involves weak activation of the mu-opioid receptors, which is more pronounced via its primary metabolite, O-desmethyltramadol (M1), while the second mechanism is related to the inhibition of neuronal reuptake of norepinephrine and serotonin [13,14]. The analgesic efficacy and safety of tramadol vary between species and appear to be enhanced when administered in combination with other drugs [15,16,17].

Despite its widespread use in veterinary practice, tramadol’s efficacy in dogs remains controversial [18,19]. Pharmacokinetic studies have demonstrated variable production of M1 in mixed-breed and Beagle dogs, which likely explains the inconsistent analgesic responses observed across individuals [15,20]. Orally administered tramadol in dogs is subject to rapid and unpredictable metabolism, and currently available evidence provides limited support for its effectiveness as a perioperative analgesic [15,19].

Given the high frequency of ovariohysterectomy and the widespread clinical use of tramadol, this surgical model has been frequently employed to assess the drug’s efficacy in postoperative pain management [21]. Nevertheless, most investigations have failed to demonstrate the superior analgesic efficacy of tramadol compared with agents such as morphine, methadone, meloxicam, or other cyclooxygenase inhibitors [22,23,24,25]. Comparable inconsistencies have been reported when tramadol was evaluated against firocoxib, celecoxib, hydrocodone bitartrate–acetaminophen, and methadone in more invasive and painful procedures, including orthopedic and vertebral surgeries [5,24,26,27,28]. Collectively, these findings indicate that tramadol is unsuitable as a sole analgesic agent or as a first-line therapy for the management of long-term orthopedic pain [21].

This research aimed to assess and contrast the analgesic properties of robenacoxib and tramadol for managing pain after femoral head and neck excision (FHNE). To measure success, the study utilized two primary indicators: the onset of initial weight-bearing (TIWB) and the achievement of final weight-bearing (TFWB). The working hypothesis posited that a multimodal approach, combining robenacoxib with tramadol, would yield greater pain relief than the administration of robenacoxib alone. 

## 2. Materials and Methods

This study involved client-owned dogs admitted to the Surgery and Obstetrics Unit of the Companion Animal Clinic, Department of Veterinary Medicine (Aristotle University of Thessaloniki, Greece) from 2022 to 2024. All included cases presented with coxofemoral pathology requiring FHNE. Prior to enrollment, informed written consent was secured from all animal owners. Preoperative assessment for each animal included a detailed clinical, orthopedic, and neurological workup, complemented by radiographic studies and a baseline clinicopathological profile (hematology and biochemistry). Criteria for exclusion from the study were:

1. Patients under 4 months of age (due to the requirements for prolonged NSAID therapy);
2. Deviations in hematological or biochemical values;
3. Concomitant lameness localized to other limbs or non-coxofemoral issues (e.g., stifle/tarsal osteoarthritis or neurological deficits);
4. Insufficient washout periods, defined as an interval between the cessation of prior therapy and study enrollment, was shorter than five elimination half-lives (5 × t_1/2_) for each specific agent, a duration necessary to ensure complete systemic clearance and avoid potential carry-over effects;
5. Unavailability for a minimum of 1-year postoperative follow-up;
6. Owner non-compliance with the re-examination schedule.

A randomization process was used to allocate dogs into two treatment cohorts (Groups A and B). Group A received postoperative robenacoxib (2 mg kg^−1^ bodyweight, sid) (Onsior; Elanco Animal Health, Basel, Switzerland) until final weight-bearing of the affected limb was achieved. Group B received tramadol in addition (5 mg kg^−1^ bodyweight, tid) (Tramal; Grunenthal GmbH, Aachen, Germany) for fifteen days.

Two primary clinical milestones were defined:

- Time of initial weight-bearing (TIWB): Identified as the first instance of postoperative limb use, regardless of the severity of lameness.
- Time of final weight-bearing (TFWB): Defined as the point at which the patient demonstrated maximum limb function (either full weight-bearing or stable, minimal residual lameness) within the 1-year follow-up period.

### 2.1. Clinical Examination

During each presentation (initial and subsequent ones), all dogs underwent a thorough physical, neurological, and orthopedic examination. Gait was evaluated via direct clinical observation and synchronized video documentation, focusing on the dog’s posture while standing, walking, and running (Table 1). To ensure inter-evaluator consistency, all assessments were performed exclusively by the primary investigator (AK). Running assessments were omitted during the initial two postoperative examinations. The first three follow-up assessments occurred on postoperative days 15, 30, and 60, followed by monthly evaluations until full weight-bearing of the affected limb was achieved. The total follow-up duration did not exceed one year.

To evaluate the coxofemoral joints, ventrodorsal and lateral radiographic projections were performed under general anesthesia at both the baseline and the conclusion of the study. During the initial phase and the subsequent three follow-ups, dogs undergoing chronic NSAID therapy were monitored via comprehensive hematological and biochemical profiles. These assessments included a complete blood count along with measurements of ALP, ALT, albumin, creatinine, and glucose. Furthermore, renal and hepatic functions were re-evaluated every three months for subjects on long-term medication.

### 2.2. Pre-Anesthetic Period

Preoperative fasting was maintained for 6–12 h based on the type of diet (canned vs. dry food), with water restricted only 2 h prior to surgery. A uniform pre-anesthetic protocol was applied to both groups, involving intramuscular dexmedetomidine (150 μg/m^2^, Dexdomitor; Orion Corporation, Espoo, Finland) and subcutaneous robenacoxib (2 mg/kg, Onsior; Elanco Animal Health, Basel, Switzerland). After intravenous catheterization, patients received tramadol (3 mg/kg, Tramal; Grunenthal GmbH, Aachen, Germany) and cephalosporin (30 mg/kg, Vifazolin; Vianex S.A., Athens, Greece) intravenously. Epidural anesthesia was performed at the aseptically prepared lumbosacral region using morphine sulfate (0.1 mg/kg, State Drug Monopoly, Famar S.A., Athens, Greece). Following induction with intravenous propofol (1 mg/kg initially, with 0.5 mg/kg increments for tracheal intubation; propofol MCT/LCT, Fresenius Kabi Hellas, Athens, Greece), anesthesia was maintained using 2% isoflurane (Isoflurane-Vet, Merial, Boehringer Ingelheim Animal Health Italia S.p.A., Milan, Italy) in oxygen (2 L/min).

### 2.3. Surgical Technique

For the FHNE procedure, animals were placed in lateral recumbency, ensuring the affected limb was positioned uppermost. Following aseptic preparation and clipping from the greater trochanter to the mid-tibia, a craniolateral approach to the coxofemoral joint was utilized [30,31]. After incising the joint capsule, a bifurcated syndesmotome was employed to detach the femoral head from the round ligament (where it was intact). Exposure and stabilization of the surgical site were achieved using Hohmann retractors. The ostectomy was executed with an oscillating saw, following a line from the medial aspect of the greater trochanter to the proximal border of the lesser trochanter [32], which remained preserved in every case. To confirm a successful excision, the joint was put through a passive range of motion; the absence of crepitus during flexion and extension indicated adequate bone removal. All procedures were performed by a single surgeon (AK). Finally, the surgical wound was closed in a standard layered fashion (muscle, subcutaneous tissue, and skin) using intradermal sutures [33].

### 2.4. Postoperative Period

Postoperative pain levels were monitored using the short-form Glasgow Composite Measure Pain Scale (CMPS-SF), a validated tool for assessing acute pain in canine patients. This scale evaluates six behavioral categories, including mobility, via thirty descriptive options ranked by pain severity. Adhering strictly to the questionnaire protocol, a total score was derived from the sum of these categories (maximum 24, or 20 if mobility was not assessable). Analgesic intervention was mandated if scores reached the established thresholds of 6/24 or 5/20. These evaluations occurred at specific intervals: 1, 2, 4, 6, 8, 20, and 24 h post-surgery (t1–t7). In cases where scores exceeded the threshold, rescue analgesia—comprising intramuscular morphine (0.3 mg/kg; State Drug Monopoly, Famar S.A., Athens, Greece) and intravenous fentanyl (3 μg/kg; Janssen Pharmaceutica NV, Beerse, Belgium)—was administered, and the subject was subsequently excluded from the study. All assessments were performed by the same trained evaluator (first author, AK) to maintain consistency and minimize inter-observer variability. The CMPS-SF scores guided the administration of additional analgesics throughout the postoperative period until the animal achieved stable pain-free function.

Patients remained hospitalized for 24 h of observation prior to discharge. Initial physiotherapy started 20 h post-op, emphasizing early limb use. Owners were instructed to strictly limit activity for a four-week duration, permitting only slow-paced leash walking while preventing running and jumping.

To promote early weight-bearing, a regimen of passive range of motion (PROM) exercises—specifically flexion and extension of the hip, stifle, and tarsus—was initiated on postoperative day one. These were performed in sets of 20–30 repetitions, 2–3 times daily. At the one-month follow-up, if limb weight-bearing remained suboptimal, the protocol was escalated to include active recovery techniques such as controlled swimming, retrograde walking, and stair climbing. Comprehensive owner education was provided to ensure compliance with these physical therapy modalities. During each re-examination, the owner’s proficiency in performing PROM exercises was evaluated to ensure technique consistency.

### 2.5. Statistical Analysis

The sample size was determined based on the total number of eligible clinical cases presented during the study period that met all inclusion criteria (convenience sampling). Data normality was assessed using the Shapiro–Wilk test. The independent samples *T*-test was used to evaluate differences in TIWB and TFWB values between the groups. The alpha value was set to 0.05. Effect size (Cohen’s d) was calculated to assess the magnitude of clinical differences. All statistical calculations were performed using JASP (Version 0.95.3) computer software.

## 3. Results

From January 2022 to June 2024, forty-two client-owned dogs were presented to the Companion Animal Clinic, but four of them (two from each treatment group) were excluded from this study and eliminated from all data analysis due to high pain scores, thus requiring additional analgesic treatment (rescue analgesia).

Finally, the clinical study involved 38 client-owned dogs, comprising 20 males and 18 females. Among them, fifteen dogs had been neutered. Sixteen dogs were of mixed breeds (mongrels), accounting for 42.1% of the total (Table 2).

The study population’s age ranged from 6 to 108 months (mean: 33.5; median: 18). In Group A, the age ranged from 6 to 90 months (mean: 29.6; median: 14), and in Group B it ranged from 6 to 108 months (mean: 37.5; median: 18).

Body weight varied between 3 and 35 kg (mean: 17.3; median: 20) across all dogs. Specifically, Group A showed a weight range of 6–30 kg (mean: 17.5; median: 20), and Group B ranged from 3 to 35 kg (mean: 17; median: 12). Indications for FHNE included coxofemoral luxation (n = 8), femoral head and/or neck fracture (n = 8), hip osteoarthritis (n = 7), hip dysplasia (n = 5), aseptic necrosis of the femoral head (n = 5), and acetabular fracture (n = 5). The preoperative duration of clinical signs (chronicity) spanned from 5 to 360 days (mean: 62.9; median: 35). In Group A, the chronicity ranged from 5 to 150 days (mean: 40.8; median: 20), and in Group B, it ranged from 10 to 360 days (mean: 84.9; median: 60).

### 3.1. Time of Weight-Bearing of Limb Subjected to FHNE

#### 3.1.1. TIWB of Limb

For the operated limb, TIWB occurred between 1 and 12 days postoperatively (mean: 6.4; median: 7). The grade of lameness ranged from 2 to 4 (mean: 2.7; median: 3). For Group A, the TIWB mean was 7.32 and the standard deviation 2.87 days, while for Group B, it was 5.53 and 3.39 days (range: 1–12 days for both).

Statistical analysis revealed that tramadol supplementation did not significantly impact TIWB, as no significant difference was observed between the two cohorts (*p* = 0.087).

#### 3.1.2. TFWB of Limb

The achievement of TFWB ranged from 48 to 82 days (mean: 63.5; median: 63).

During this period, all dogs received postoperative NSAIDs and physiotherapy.

The TFWB for Group A ranged from 50 to 82 days (mean 64.16, median 62) and for Group B from 48 to 74 (mean 62.95, median 65).

The addition of tramadol did not significantly influence the TFWB of the affected limb (*p* = 0.659). This is further supported by the negligible effect size (*d* = 0.144), indicating that the recovery time to final weight-bearing was essentially equivalent between the two groups regardless of tramadol administration. Regarding the safety profile of the treatments, no adverse effects, such as gastrointestinal disturbances (vomiting, diarrhea, or anorexia) or excessive sedation, were observed in either group throughout the study period. All dogs tolerated the oral administration of robenacoxib and the combination of robenacoxib with tramadol without requiring any dose adjustments or medical intervention.

### 3.2. Glasgow Composite Measure Pain Scale

Postoperative pain was monitored at seven time points (t_1_ to t_7_) using the CMPS-SF. For the 38 dogs that completed the study, mean pain scores remained well below the rescue analgesia threshold, ranging from 2.13 at t_1_ to a peak of 3.84 at t_6_ (Table 3). Statistical analysis showed no significant differences in pain scores between Group A and Group B at any time point (*p* > 0.05). Four additional dogs (two from each group) were excluded from the final analysis as they required rescue analgesia at t_2_ (n = 2) and t_3_ (n = 2) after exceeding the threshold (scores > 6).

## 4. Discussion

This blinded clinical study sought to evaluate whether the addition of tramadol to a robenacoxib regimen provides superior limb function recovery compared to robenacoxib monotherapy in dogs following FHNE. Four dogs required rescue analgesia in the immediate postoperative period and were therefore excluded from this study. The requirement for additional analgesic treatment may indicate inadequate analgesic efficacy of the selected drugs, or it may reflect the use of a relatively low Glasgow Composite Measure Pain Scale threshold for administering rescue analgesia.

The choice of robenacoxib as the baseline NSAID for all animals in this study is supported by its proven efficacy and safety profile in long-term canine orthopedic care [34,35,36]. While FHNE recovery is an acute postoperative process, it often transitions into a chronic management phase similar to osteoarthritis, where robenacoxib has been extensively validated.

Research conducted by Edamura et al. (2012) [37] established that robenacoxib is non-inferior to carprofen in managing chronic lameness and pain over a 12-week period, matching the timeline observed in our study for TFWB. Furthermore, the high safety margin reported by King et al. (2011) [38]—who observed no adverse gastrointestinal or renal effects during 6 months of continuous administration—justifies its use throughout the intensive physiotherapy phase required after FHNE.

A key pharmacological advantage of robenacoxib is its tissue selectivity. Unlike other NSAIDs, it maintains a short systemic half-life while persisting at the site of inflammation [39,40,41]. In the context of our study, this characteristic likely provided sustained analgesia within the pseudoarthrosis site, facilitating the early limb use (TIWB) and long-term remodeling (TFWB) observed in both groups. The fact that TFWB reached a mean of approximately 63 days across both groups aligns with the “plateau” of recovery often seen in long-term robenacoxib trials, where the drug maintains comfort during the critical window of soft-tissue healing and muscle strengthening.

As a synthetic opioid analgesic, tramadol is frequently employed across both human and veterinary clinical practices for pain management. During the last decade, its use has expanded beyond surgical pain management to conditions such as osteoarthritis. However, its efficacy remains controversial. Several studies have compared tramadol with other analgesics across different surgical procedures, particularly ovariohysterectomy [19]. In the context of anesthetic premedication, tramadol was associated with a lower incidence of rescue analgesia requirements compared to butorphanol and buprenorphine, though the findings remained statistically equivocal [19,23,42]. Comparison with morphine has shown no significant analgesic difference [22,43,44]. Donati et al. (2021) [19] observed that an increased number of dogs required rescue analgesia when treated with tramadol compared to NSAIDs, suggesting reduced efficacy. Budsberg et al. (2018) [45] reported the role of tramadol in the management of stifle and elbow osteoarthritis, equating it with placebo therapy. However, the small sample sizes and limited number of studies make these conclusions uncertain.

The pharmacokinetics of tramadol in the canine species are highly variable. Dogs are known to be “poor producers” of the O-desmethyltramadol (M1) metabolite, which is responsible for the majority of the μ-opioid receptor activity [13,15]. Our chosen dosage of 5 mg/kg TID follows common clinical guidelines; however, it is possible that this oral regimen failed to reach therapeutic plasma concentrations of the active metabolite. The results of this study are therefore specific to this oral dosing frequency and may not reflect the efficacy of tramadol administered via other routes or at higher dosages.

Few studies have examined tramadol in orthopedic procedures, and even fewer have directly compared its effectiveness with NSAIDs. Orthopedic surgeries are painful procedures and typically require multimodal analgesia for additive or synergistic effects, particularly in the postoperative period. The combination of opioids and NSAIDs is often considered more beneficial than administering either drug alone in both human and veterinary medicine [46,47,48,49].

Yazbek and Fantoni (2005) [14] retrospectively compared tramadol and flunixin meglumine in orthopedic surgeries, administering acepromazine mixed with either tramadol or flunixin as pre-anesthetic medication in two groups of fifteen dogs. They suggested the effective role of tramadol in pre-emptive analgesia and in improved recovery. In a study involving canine patients after tibial plateau leveling osteotomy (TPLO), Benitez et al. (2015) [26] compared the postoperative analgesic efficacy of tramadol against a hydrocodone–acetaminophen combination. They did not detect any significant difference in their efficacy, except at one time point, two hours after the second dose, where the hydrocodone–acetaminophen group had a lower pain score than the median. In osteosynthesis procedures, tramadol premedication produced longer-lasting analgesia than butorphanol [42]. Across these studies, the assessment of pain was conducted with different pain scales, namely the Visual Analog Scale and modified Glasgow Composite Measure Pain Scale, and the effect of the analgesic drugs on limb function was not examined.

Davila et al. (2013) [5] retrospectively evaluated the clinical efficacy of tramadol, firocoxib, and a tramadol–firocoxib combination using three groups of client-owned dogs. Pain was assessed using the short-form Glasgow Composite Measure Pain Scale, serum cortisol concentrations, and a pressure platform gait analysis. The results indicated that tramadol is not effective as an analgesic monotherapy and cannot provide any benefit in weight-bearing postoperatively. While prior research into multimodal protocols suggested that tramadol might influence TIWB, its impact on TFWB remains statistically negligible [29].

In this study, both groups received the same pre-anesthetic regimen. Differentiation was only introduced in postoperative medication to eliminate the effect of deviations from the anesthetic protocol. The assessment of TIWB did not detect any statistically significant difference between the two groups, despite the two-unit difference in the medians. According to the statistical results of TFWB, there is a three-unit difference in the medians, but this is not statistically significant. Postoperative data revealed that neither TIWB nor TFWB improved significantly when tramadol was added to a robenacoxib regimen. Consequently, it can be inferred that tramadol fails to provide supplemental analgesia beyond the effects already achieved by robenacoxib as a standalone treatment. The lack of tramadol’s anti-inflammatory efficacy could be the reason for its limited benefit in limb function postoperatively [50].

However, the duration of lameness renders our evidence uncertain. A positive correlation between disease chronicity and final weight-bearing of the affected limb has been established in the literature [51,52,53,54,55,56,57]. In this study, Group A had chronicity ranging from 5 to 150 days (mean: 40.8; median: 20), whereas in Group B it ranged from 10 to 360 days (mean: 84.9; median: 60). The observed discrepancy in the duration of underlying disease between groups is notable. Chronic coxofemoral pathology is frequently associated with compensatory muscle atrophy and central sensitization. The longer disease duration in Group B may have resulted in more profound preoperative debilitation, potentially masking the analgesic efficacy of tramadol. Future studies should consider stratified randomization based on disease chronicity.

The inclusion of a control group would have helped to clarify this effect, but it would have been unethical to perform orthopedic surgery using tramadol as monotherapy postoperatively.

In the early postoperative period, TIWB showed a mean difference of approximately 1.8 days between the two groups. Although this did not reach the threshold for statistical significance (*p* = 0.087), the calculated effect size of 0.570 indicates a potentially moderate clinical effect. This suggests that this study may have been underpowered to detect a statistically significant benefit of tramadol. In clinical practice, a Cohen’s *d* of 0.570 is often considered a “medium” effect, implying that while the results are not mathematically definitive, there is a visible trend toward faster initial recovery in animals receiving the multimodal analgesic protocol. Unlike the p-value, which is highly dependent on sample size, the effect size (*d* = 0.570) provides a sample-size-independent estimate of the magnitude of the difference, pointing towards a clinically relevant improvement in the early postoperative period.

We acknowledge that the study’s sample size (n = 19 per group) may limit the ability to detect such differences at a *p* < 0.05 level. However, rather than relying on retrospective power calculations—which can be misleading [58]—we emphasize the magnitude of the observed effect. The “medium” effect size and the numerical trend in TIWB suggest a potential clinical benefit for the multimodal protocol, implying that the absence of statistical significance should not be equated with a definitive lack of clinical efficacy. To provide a roadmap for future clinical trials, a power analysis based on the observed effect size of *d* = 0.570 indicates that approximately 50 dogs per group (N = 100 total) would be required to achieve a statistical power of 0.80 at a significance level of *p* = 0.05. This highlights that while our study provides evidence of a clinical trend, larger multi-center cohorts may be necessary to confirm the statistical superiority of this multimodal protocol for TIWB.

In contrast, TFWB showed no meaningful divergence between the groups (*p* = 0.659). The negligible effect size (*d* = 0.144) confirms that the long-term functional outcome was essentially identical regardless of the initial analgesic intervention. This suggests that while tramadol may influence the comfort levels required for the first steps (TIWB), the total duration required for full limb function is likely governed by biological healing processes and the standardized physiotherapy protocol rather than the specific postoperative analgesic used.

A potential limitation of this study is the continuous administration of robenacoxib throughout the experimental period, which may have masked the clinical effects of tramadol. Robenacoxib, as a highly selective COX-2 inhibitor, provides potent anti-inflammatory and analgesic effects at the site of inflammation. This baseline analgesia could have created a “ceiling effect,” making it difficult to discern the incremental benefit of tramadol. If tramadol were administered alone or alongside a non-analgesic substance, the observed opioid-mediated effects might have been more pronounced. This interaction suggests that while multimodal protocols are clinically preferred, they can complicate the isolation of a single drug’s efficacy in a research setting.

Finally, while all owners were provided with a standardized physiotherapy protocol, compliance was not objectively monitored. Variations in the intensity and frequency of home-based exercises represent a potential confounding variable, as recovery following FHNE is heavily dependent on consistent physical rehabilitation.

## 5. Conclusions

In conclusion, the addition of oral tramadol to a robenacoxib regimen following FHNE in dogs did not result in a statistically significant improvement in functional limb recovery. However, the observed moderate effect size (*d* = 0.570) for initial weight-bearing suggests a clinically interesting trend toward faster early recovery in the multimodal group. While these findings suggest a potential clinical benefit, the study may have been underpowered to reach statistical significance.

Consequently, these findings should be interpreted as a potential clinical direction rather than a definitive advantage. Future multi-center studies with larger cohorts (approximately 50 dogs per group) are warranted to further elucidate the specific analgesic contribution of tramadol and confirm whether this observed trend translates into a statistically significant benefit in orthopedic recovery.

## Figures and Tables

**Table 1:** Lameness scale [29].

Degree of Lameness	Limb’s Weight-Bearing				Characterization of Lameness
	Description	Stance	Walk	Run	
0	Full (normal) weight-bearing				Absence
1	Partial weight-bearing: hardly visible				Light
2	Partial weight-bearing: easily visible				Mild
3	No weight-bearing: intermittent, sporadic (≤1:5) *				Moderate
4	No weight-bearing: intermittent, frequent (>1:5) *				Severe
5	No weight-bearing: continuous				Not functional
Degree of lameness = (S + W + R)/3					

*: limb lift frequency per five steps.

**Table 2:** Distribution of dog breeds submitted to femoral head and neck excision.

Breed	Number of Dogs
Mongrel	16
Golden Retriever	7
German Shepherd	5
Poodle	2
Shih Tzu	2
Pomeranian	2
Yorkshire	2
Rottweiler	1
Cane Corso	1

**Table 3:** Mean CMPS-SF pain scores at each postoperative time point (n = 38).

Time Point	Mean Score	Range (Min–Max)
t_1_ (1 h)	2.13	2–4
t_2_ (2 h)	2.26	2–4
t_3_ (4 h)	2.34	2–5
t_4_ (6 h)	2.42	2–5
t_5_ (8 h)	3.63	3–5
t_6_ (20 h)	3.84	3–5
t_7_ (24 h)	3.42	2–5

## Data Availability

The original contributions presented in this study are included in the article. Further inquiries can be directed to the corresponding author.
